# Supplementary figures and images for: Relationships between total reserve and financial indicators of Bangladesh: Application of generalized additive model
Source: PLoS One. 2023 Apr 7;18(4):e0284179. doi: 10.1371/journal.pone.0284179 (PMC10081751; doi:10.1371/journal.pone.0284179)

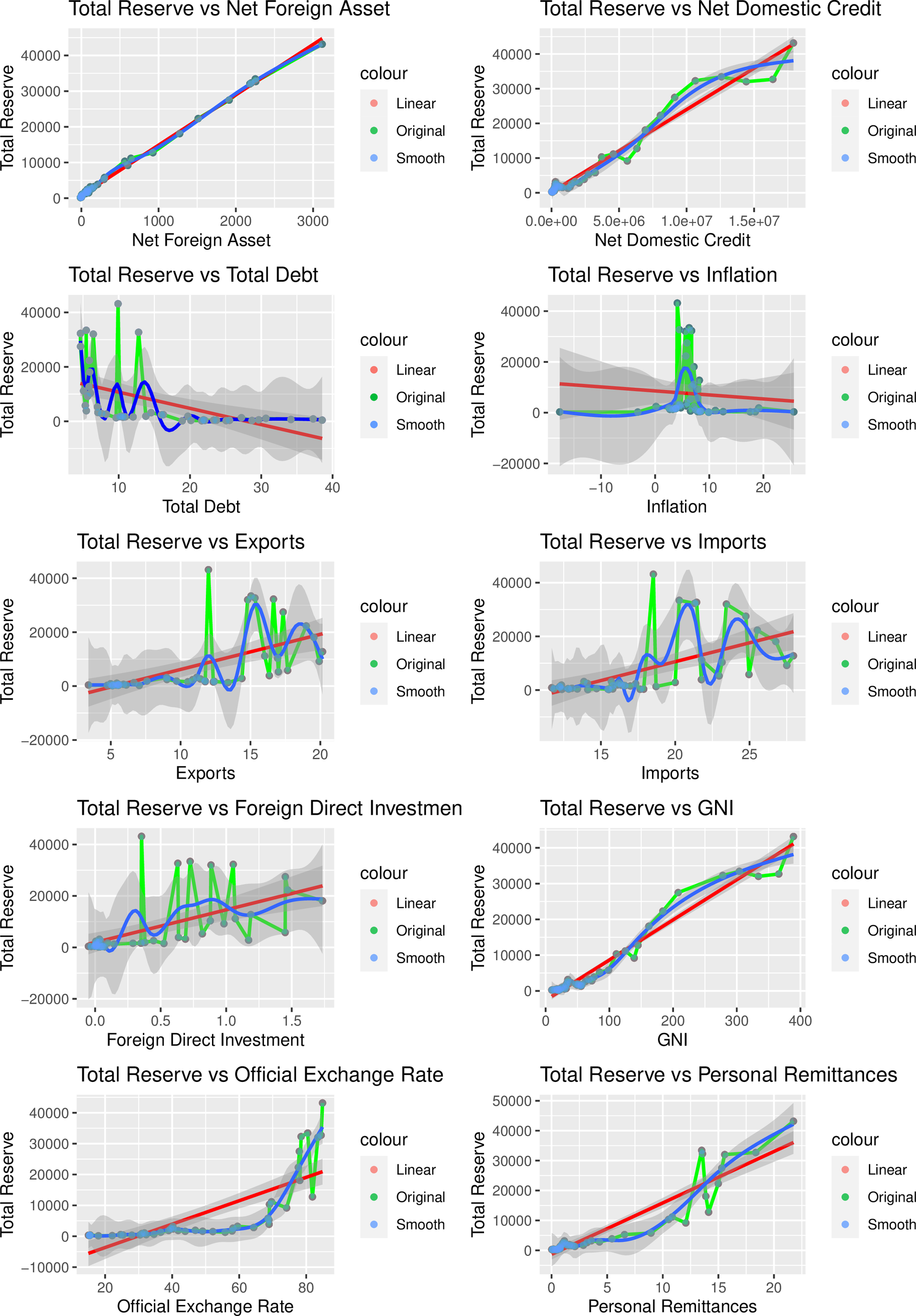

Supplement: S1 Fig — (TIF) [file pone.0284179.s001.tif]
