# Supplementary material for: Relationships between total reserve and financial indicators of Bangladesh: Application of generalized additive model
Source: PLoS One. 2023 Apr 7;18(4):e0284179. doi: 10.1371/journal.pone.0284179 (PMC10081751; doi:10.1371/journal.pone.0284179)
Supplement: Attachment — Submitted filename: Response to Reviewers.docx [file pone.0284179.s002.docx]

**Author responses to the review comments:**

We would like to express our sincere gratitude to the three reviewers and the Academic Editor for their valuable comments. We have considered all the comments made by the reviewers and thoroughly revised and formatted the manuscript accordingly. A detailed response to each of the comments is provided in the table below:

| **Academic Editor comments:** | **Response** | **Note** |
| --- | --- | --- |
| Please include the following items when submitting your revised manuscript:  A rebuttal letter that responds to each point raised by the academic editor and reviewer(s). You should upload this letter as a separate file labeled 'Response to Reviewers'.  A marked-up copy of your manuscript that highlights changes made to the original version. You should upload this as a separate file labeled 'Revised Manuscript with Track Changes'.  An unmarked version of your revised paper without tracked changes. You should upload this as a separate file labeled 'Manuscript'.  If you would like to make changes to your financial disclosure, please include your updated statement in your cover letter. Guidelines for resubmitting your figure files are available below the reviewer comments at the end of this letter. | Thank you very much. The required files are submitted through the submission system.  We include all required information in the cover letter. | Revised texts are in red color. |
| **Journal Requirements:** | **Response** | **Note** |
| When submitting your revision, we need you to address these additional requirements.  1. Please ensure that your manuscript meets PLOS ONE's style requirements, including those for file naming. The PLOS ONE style templates can be found at  https://journals.plos.org/plosone/s/file?id=wjVg/PLOSOne_formatting_sample_main_body.pdf and  https://journals.plos.org/plosone/s/file?id=ba62/PLOSOne_formatting_sample_title_authors_affiliations.pdf | Many thanks. We revised the manuscript following the PLOS ONE style. |  |
| 2. In your Data Availability statement, you have not specified where the minimal data set underlying the results described in your manuscript can be found. PLOS defines a study's minimal data set as the underlying data used to reach the conclusions drawn in the manuscript and any additional data required to replicate the reported study findings in their entirety. All PLOS journals require that the minimal data set be made fully available. For more information about our data policy, please see http://journals.plos.org/plosone/s/data-availability.  Upon re-submitting your revised manuscript, please upload your study’s minimal underlying data set as either Supporting Information files or to a stable, public repository and include the relevant URLs, DOIs, or accession numbers within your revised cover letter. For a list of acceptable repositories, please see http://journals.plos.org/plosone/s/data-availability#loc-recommended-repositories. Any potentially identifying patient information must be fully anonymized.  Important: If there are ethical or legal restrictions to sharing your data publicly, please explain these restrictions in detail. Please see our guidelines for more information on what we consider unacceptable restrictions to publicly sharing data: http://journals.plos.org/plosone/s/data-availability#loc-unacceptable-data-access-restrictions. Note that it is not acceptable for the authors to be the sole named individuals responsible for ensuring data access.  We will update your Data Availability statement to reflect the information you provide in your cover letter. | Thanks. We update the data availability statement.  The data is freely available on World Development Indicators of World Bank database. Data can be access through the link: https://data.worldbank.org/country/BD | Revised texts are in red color.  Page: 20 |
| 3. We note you have included a table to which you do not refer in the text of your manuscript. Please ensure that you refer to Table 2 in your text; if accepted, production will need this reference to link the reader to the Table. | Thanks. We refer Table 2 in the texts. | Revised texts are in red color.  Page: 12 |
| 4. Please include captions for your Supporting Information files at the end of your manuscript, and update any in-text citations to match accordingly. Please see our Supporting Information guidelines for more information: http://journals.plos.org/plosone/s/supporting-information. | Thank you very much. We add the caption of Supporting Information files at the end of the manuscript and update the citation in texts. | Revised texts are in red color.  Page: 19 |
| **Reviewer 1 comments:** | **Response** | **Note** |
| It is unclear what is the motivation of this study. Indeed, this study has identified some anomalies (net outcome), which I have identified in my summary above. One can find so many things through big data analytics, which are sometimes interesting. However, in academic study, there are two broad approaches. First, the study could identify a research question based on a debate in the literature, identify the knowledge gap, and draw a hypothesis to test. Second, based on conjecture or general observation, do empirical tests, and find results that can be explained deeply in the context of theory and broader literature. I see neither approach in this study. | Thank you very much for your comments. We revise the manuscript incorporating the research gap, revised the objectives, and updated the methodology and result sections. | Revised texts are in red color.  Page: 1-19 |
| **Reviewer 2 comments:** | **Response** | **Note** |
| Dear authors,  I'd like to congratulate you and your team on your excellent research work in your paper submitted for publication in this prestigious journal. The topic is very interesting, and I enjoyed it. I would like to thank you for your efforts in presenting your research work in such a professional manner. However, before your work is recommended or accepted, a few comments must be included/ addressed to improve the quality of your work as well as for future publication in this reputable journal. I have the following observations, questions, and comments that may help to improve your work. The authors must modify the following points in great detail.  1. In the abstract, please include 2-3 special quantitative achievements from the findings of this study in the context of the environment by combining the research objectives and problems. Please limit your abstract to 250 words. Check spellings for many words that are misspelt or written in haste. | Thank you very much for your comments and feedback. We believe that it helps to improve the quality of the manuscript.  As we know, POLS ONE accepts up to 500 words in abstract. In our case, it consists of 250 words. We also check the spelling. | Revised texts are in red color.  Page: 1-2 |
| 2. The introduction section needs a few more sentences to strengthen the article, and please include the research problem, objective, and novelty in the last paragraph of the Introduction section. | Thanks. We revised the Introduction section. | Revised texts are in red color.  Page: 3-5 |
| 3. Include a few more sentences at the beginning of the introduction explaining your paper's contribution to the environment, climate change impact, and sustainability, as well as your attempts to deal with or present solutions to a specific problem/s and your unique contribution with this research paper. | Thank you very much. Actually, this manuscript does not consider the climate variables, however, we revise the manuscript and tried to link economic growth and economic development. | Revised texts are in red color.  Page: 4-5 |
| 4. Please also present the methodology section in a concise graphical format. | Thank. We add a figure to present the research framework. | Revised texts are in red color.  Page: 5-6 |
| 5. The literature review section is very weak; please revise it. | Thanks. We revised the manuscript. | Revised texts are in red color.  Page: 2-5 |
| 6. Please present your literature review in the form of a SmartArt chart. | Thank you very much. We add a flowchart in the Methodology section. | Revised texts are in red color.  Page: 5-6 |
| 7. Just after the Methodology, please mention the societal benefits of your research in terms of evaluating its key determinant. | Thanks. We add it in the Methodology section. | Revised texts are in red color.  Page: 5 |
| 8. In 500-750 words, explain research problems, solutions, and the theoretical contribution of your study in the "Results" section. | Thanks. Actually, it is an applied work. The authors tried to find the most appropriate model to predict the total reserve. The result section is revised as per your suggestion. | Revised texts are in red color.  Page: 9, 17 |
| 9. Please include graphical presentations of your findings. | Thanks. We add a figure to present the final findings of this manuscript. | Revised texts are in red color.  Page: 17 |
| 10. Describe why you placed this study in a separate section of "Policy Suggestions" just before the section of "Conclusions."  I found that the literature section is a little weak, shift your study a little more towards environment friendly and sustainability, therefore it requires more studies to be reviewed therefore I suggest you to include the following work:  https://doi.org/10.1016/j.jclepro.2021.128585  https://doi.org/10.1016/j.jclepro.2021.128109  https://doi.org/10.1016/j.heliyon.2021.e06952  https://doi.org/10.1007/s11356-022-20567-6  https://doi.org/10.1177/09763996211041215  https://doi.org/10.1007/s11356-021-15421-0  https://doi.org/10.1007/s11356-021-14745-1  https://doi.org/10.1007/s11356-021-13441-4  https://doi.org/10.1007/s10668-021-01418-9  https://doi.org/10.1016/j.techfore.2022.121524  https://doi.org/10.1016/j.resourpol.2022.102612  I think above all studies will make this study more relevant in bridging the gap with literature.  Looking forward for your revised submission. | Thanks. We revised the manuscript in light of the suggested papers and cite them. | Revised texts are in red color.  Page: 3-5 |
| **Reviewer 3 comments:** | **Response** | **Note** |
| The topic is interesting. However, the paper looks like a technical report more than a research article. The manuscript has not been written up to the standards of the journal. I am not sure about the goal of the paper. It seems that mathematical narration is fine. The paper is a statistical practice without a well-justified motivation. Motivation is weak. The selection of variables is arbitrary rather than theoretical and empirical consideration. The paper cannot persuade the readers due to weak evidences and arguments in designing research model. Overall, I am afraid, I cannot recommend it for publication in PloS ONE. | Thank you very much for your comment and feedback. We revise the motivation and objectives of this manuscript. We hope the revised version will satisfy your expectation. | Revised texts are in red color.  Page: 1-19 |

Finally, the revised manuscript has been produced following the valuable comments and suggestions of the reviewers. Once again, we would like to thank the reviewers for their sincere dedication, professional insights, and earnest cooperation in reviewing the manuscript.
