# Supplementary material for: Relationships between total reserve and financial indicators of Bangladesh: Application of generalized additive model
Source: PLoS One. 2023 Apr 7;18(4):e0284179. doi: 10.1371/journal.pone.0284179 (PMC10081751; doi:10.1371/journal.pone.0284179)
Supplement: Attachment — Submitted filename: Response to Reviewers.docx [file pone.0284179.s003.docx]

**Author responses to the review comments:**

We would like to express our sincere gratitude to the three reviewers and the Academic Editor for their valuable comments. We have considered all the comments made by the reviewers and thoroughly revised and formatted the manuscript accordingly. A detailed response to each of the comments is provided in the table below:

| **Academic Editor comments:** | **Response** | **Note** |
| --- | --- | --- |
| Please include the following items when submitting your revised manuscript:  A rebuttal letter that responds to each point raised by the academic editor and reviewer(s). You should upload this letter as a separate file labeled 'Response to Reviewers'.  A marked-up copy of your manuscript that highlights changes made to the original version. You should upload this as a separate file labeled 'Revised Manuscript with Track Changes'.  An unmarked version of your revised paper without tracked changes. You should upload this as a separate file labeled 'Manuscript'.  If you would like to make changes to your financial disclosure, please include your updated statement in your cover letter. Guidelines for resubmitting your figure files are available below the reviewer comments at the end of this letter. | Thank you very much. The required files are submitted through the submission system.  We include all required information in the cover letter. | Revised texts are in red color. |
| **Editor Comments:** | **Response** | **Note** |
| Dear Authors,  I find your work has a very minimal contribution to the introduction, literature, and methodology part. Please consider all outstanding revision requests from all reviewers, including the reviewers who recommended rejection. As, I don’t think your contribution is good enough to be published in a very high standard journal like the Plos One.  You can respond to the comments in this thread and resubmit the revised manuscript. We encourage you to submit your revised manuscript with tracked changes to facilitate the review. | Many thanks. We revised the manuscript as per the review comments. | Revised texts are in red color.  Page: 1-19 |
| **Reviewer 2 comments:** | **Response** | **Note** |
| Dear Authors,  I would like to congratulate you and your team for doing such a good research work in your submitted paper. Topic is very interesting and I liked the topic and appreciate your efforts to present your revised research work in such a nice manner. I am satisfied from your efforts you employed in the revision and I found all my suggested comments have been incorporated or addressed perfectly. Therefore, I strongly recommended this article for acceptance for further publication in this reputed journal without any more changes.  The revised version of the paper looks perfect, in the section of the literature review the very relevant, connected, and updated with new references.  Methodology mentions this research's socio-economic benefits in evaluating its crucial determinant.  The section on results in the revised version explains all of the tables more briefly, and the explanations for each table in the "Results" sections, define more visibility of empirical results.  In the revised version after adding about 150 words to the conclusion section related to policy implications explaining and connecting the future scope of your research study, any limitations encountered while conducting your research, and the procedure for removing research limitations.  The revised version communicates the introduction which provides helpful information about the intended topic; however, it needed to be revised to make it meaningful and the authors are successfully able to revise it. Indeed, the research GAP is now well explained in a scientific way. Therefore, the research can be recommended for publication without any more modification in its the current form which provides sufficient justification and arguments. | Thank you very much for your positive as well as detailed comments.  We are also thankful to you for recommending the manuscript for publication without any more modification in its current form. |  |
| **Reviewer 3 comments:** | **Response** | **Note** |
| The revised version of the manuscript seems to contain the previous mistakes and shortcomings event though the author attempt to the response my queries. In general, the paper looks like a report paper or a project to test out existing empirical methods on different quantitative techniques rather than an academic paper. This paper does not meet the requirements for a scientific article. The motivation is still not strong enough. Literature review section is missing. Theoretical background of selection of the variables is missing. Therefore sorry for inconvenience and harsh decision from my side. I do NOT recommend this work for publication in as a prestigious journal as PloS ONE. | Thank you very much for your comments and feedback. We believe that your negative comments help to improve the quality of the manuscript.  The objective of this paper is to identify the nature of the relationship and influence of economic indicators on the total reserve of Bangladesh using a suitable statistical model.  From our previous experience of publishing papers in PloS ONE as well as the guidelines of this journal, we format this paper.  We revise the motivation of this study.  Basically, we merge the Background and Literature review following many of the published papers in PloS ONE. As per your comments, we add the literature review section separately.  The selection of carraraites is based on the existing literature review, data availability, and self-efficacy of the authors. | Revised texts are in red color.  Page: 4-6 |

Finally, the revised manuscript has been produced following the valuable comments and suggestions of the reviewers. Once again, we would like to thank the reviewers for their sincere dedication, professional insights, and earnest cooperation in reviewing the manuscript.
